# Integrative Multi-Omics Profiling of Rhabdomyosarcoma Subtypes Reveals Distinct Molecular Pathways and Biomarker Signatures

**DOI:** 10.3390/cells14141115

**Published:** 2025-07-20

**Authors:** Aya Osama, Ahmed Karam, Abdelrahman Atef, Menna Arafat, Rahma W. Afifi, Maha Mokhtar, Taghreed Khaled Abdelmoneim, Asmaa Ramzy, Enas El Nadi, Asmaa Salama, Emad Elzayat, Sameh Magdeldin

**Affiliations:** 1 Proteomics and Metabolomics Unit, Basic Research Department, Children’s Cancer Hospital (CCHE-57357), Cairo 57357, Egypt; aya.osama@57357.org (A.O.); ahmed.karam@57357.org (A.K.); abdelrahmanatef2717@gmail.com (A.A.); rahmawaleed2308@gmail.com (R.W.A.); maha.muawad@57357.org (M.M.); taghreed.khaled@57357.org (T.K.A.); asmaa.ramzy@57357.org (A.R.); 2 Faculty of Medicine, Mansoura University, Mansoura 35516, Egypt; studentmennaarafat@gmail.com; 3 Radio Diagnosis Department, Children’s Cancer Hospital (CCHE-57357), Cairo 57357, Egypt; inas.mohsen@57357.org; 4 Faculty of Medicine, Beni-Suef University, Beni-Suif 62521, Egypt; 5 Pathology Department, Children’s Cancer Hospital (CCHE-57357), Cairo 57357, Egypt; asmaa.salama@57357.org; 6 National Cancer Institute, Cairo University, Cairo 11562, Egypt; 7 Biotechnology Department, Faculty of Science, Cairo University, Cairo 11562, Egypt; elzayatem@sci.cu.edu.eg; 8 Department of Physiology, Faculty of Veterinary Medicine, Suez Canal University, Ismailia 41522, Egypt

**Keywords:** rhabdomyosarcoma, embryonal RMS (ERMS), alveolar RMS (ARMS), proteomics, metabolomics, multi-omics integration

## Abstract

Rhabdomyosarcoma (RMS), the most common pediatric soft tissue sarcoma, comprises embryonal (ERMS) and alveolar (ARMS) subtypes with distinct histopathological features, clinical outcomes, and therapeutic responses. To better characterize their molecular distinctions, we performed untargeted plasma proteomics and metabolomics profiling in children with ERMS (*n* = 18), ARMS (*n* = 17), and matched healthy controls (*n* = 18). Differential expression, functional enrichment (GO, KEGG, RaMP-DB), co-expression network analysis (WGCNA/WMCNA), and multi-omics integration (DIABLO, MOFA) revealed distinct molecular signatures for each subtype. ARMS displayed elevated oncogenic and stemness-associated proteins (e.g., cyclin E1, FAP, myotrophin) and metabolites involved in lipid transport, fatty acid metabolism, and polyamine biosynthesis. In contrast, ERMS was enriched in immune-related and myogenic proteins (e.g., myosin-9, SAA2, S100A11) and metabolites linked to glutamate/glycine metabolism and redox homeostasis. Pathway analyses highlighted subtype-specific activation of PI3K-Akt and Hippo signaling in ARMS and immune and coagulation pathways in ERMS. Additionally, the proteomics and metabolomics datasets showed association with clinical parameters, including disease stage, lymph node involvement, and age, demonstrating clear molecular discrimination consistent with clinical observation. Co-expression networks and integrative analyses further reinforced these distinctions, uncovering coordinated protein–metabolite modules. Our findings reveal novel, subtype-specific molecular programs in RMS and propose candidate biomarkers and pathways that may guide precision diagnostics and therapeutic targeting in pediatric sarcomas.

## 1. Introduction

Sarcomas are a heterogeneous group of rare cancers arising from mesenchymal cells that form connective tissues such as bone, muscle, and cartilage. With over 70 subtypes based on histological and molecular characteristics, sarcomas represent less than 1% of adult cancers but account for a significant proportion of pediatric cases, including rhabdomyosarcoma (RMS), one of the most aggressive soft tissue sarcomas in children [1,2]. RMS is characterized by high metastatic potential and poor prognosis, highlighting the need for a deeper understanding of its molecular basis to develop targeted therapies [3].

RMS originates from undifferentiated mesenchymal cells with the potential to form skeletal muscle. It can develop in various anatomical sites, including the head and neck, genitourinary tract, and extremities, each presenting distinct clinical and molecular features [4]. The two primary subtypes of RMS are alveolar RMS (ARMS) and embryonal RMS (ERMS), which exhibit distinct clinical, molecular, and histopathological characteristics. ARMS is generally more aggressive and often linked to specific chromosomal translocations, such as PAX3-FOXO1, though not all ARMS cases exhibit this fusion gene [2]. This absence of fusion genes highlights the molecular heterogeneity within ARMS and points to alternative mechanisms of tumorigenesis. Conversely, ERMS is associated with a broader genetic profile and a relatively more favorable prognosis. Current diagnostic approaches for RMS depend mainly on imaging techniques and pathological evaluations [5]. Although methods like ultrasonography, CT, and MRI are used to assess tumor burden and treatment response, they have limitations, particularly in detecting small lesions and monitoring molecular changes.

Recent advancements in multi-omics technologies, such as proteomics and metabolomics, offer transformative opportunities to understand the molecular landscape of RMS. Proteomics enables the identification of dysregulated proteins, while metabolomics highlights altered metabolic pathways. Together, both provide a thorough understanding of the tumor development, progression, and related biological processes [6,7]. Integrating these approaches is particularly valuable for uncovering subtype-specific differences between ARMS and ERMS. Although proteomics and metabolomics have been applied to RMS, most studies have been limited to in vitro or in vivo models rather than patient-derived samples, restricting their clinical applicability [8]. Expanding these investigations to patient-derived plasma samples holds the potential to enhance our understanding of RMS and its subtypes, facilitating the possible identification of clinically relevant biomarkers for early detection and precision therapy.

The advancement of cutting-edge ‘multi-omics’ aims to unravel the molecular aberrations that underlie the onset and progression of childhood RMS. In this study, we utilized a multi-omics approach to explore the molecular differences between ARMS and ERMS. By analyzing plasma samples from RMS patients, we aimed to uncover key proteins and metabolites differentiating these subtypes and identify possible novel biomarkers for disease progression and therapeutic response. This comprehensive approach provides an opportunity to discover the molecular pathways driving RMS and contributes to the development of more precise and effective treatment strategies.

## 2. Materials and Methods

### 2.1. Human Subjects

Plasma samples were retrospectively collected from the biorepository at Children’s Cancer Hospital 57357, Egypt (CCHE-57357). Written informed consent was obtained from the guardians of all participants, and the study was approved by the Institutional Research Board (IRB-CCHE-57357-45-2023). A cohort of 35 patients, initially diagnosed with RMS, was included in this study. This cohort comprised 17 patients with ARMS and 18 with ERMS; all were sampled before receiving chemotherapy or radiotherapy. Subtypes were determined through histopathological examination following the COG-ARST2031 guidelines adopted at the CCHE-57357 [9]. Inclusion criteria for the study consisted of patients under 18 years of age diagnosed with RMS in the head and neck region (Parameningeal: Nasopharynx, Paranasal Sinuses, Middle Ear, Mastoid Region, Pterygoid-Infratemporal Fossae), categorized as intermediate or high risk. Tumor classification variables included size (b > 5 cm or a = ≤ 5 cm), lymph node involvement (clinically involved or not clinically involved), stage (III or IV), and surgical group classification (group 3, group 4, or group 1). For the healthy control group, plasma samples were collected from 18 individuals with no prior history of disease in the past six months and normal complete blood count (CBC) results. All collected samples were stored at −80 °C until use.

### 2.2. Proteomics Shotgun Analysis

#### 2.2.1. Plasma Albumin Depletion

Plasma samples were quantified using the Pierce Detergent Compatible Bradford Assay Kit (Catalog # 23246, Thermo Fisher Scientific, Waltham, MA, USA). Samples were diluted threefold with Milli-Q water, and albumin was depleted using the Pierce Albumin Depletion Kit (Catalog # 85160, Thermo Fisher Scientific, Waltham, MA, USA) as previously described [10]. In this approach, 200 µL of albumin depletion resin solution was used to bind to 2 mg of human albumin. The resin was added to the spin column and centrifuged at 12,000× *g* for 1 min at room temperature. In a new collection tube, 75 µL of the diluted plasma sample was applied to the spin column and centrifuged at 12,000× *g* for 1 min. In this step, the flow-through represents the albumin-depleted plasma samples. To release any unbound proteins on the column, the spin column was washed with 50 μL of the Pierce binding/wash buffer (25 mM Tris, 75 mM NaCl, pH 7.5); this step was repeated twice. The steps of the washing were combined with the albumin-depleted plasma samples. Finally, for the elution of the bound albumin, 200 μL NaCl was added to a new collection tube. The total amount of protein was quantified by Bradford assay using Pierce Detergent Compatible Bradford Assay Kit (Catalog # 23246, Thermo Fisher Scientific, Waltham, MA, USA). To further confirm the depletion efficiency, depleted plasma samples with washes and albumin-eluted fractions of two random samples were analyzed by SDS-PAGE. A mass of 5 μg of the sample was mixed with 2× Laemmli sample buffer with 5% β-mercaptoethanol, heated at 95 °C for 15 min, and electrophoresed on a 12.5% SDS-PAGE gel at 125 V for 90 min. The gel was stained with Silver Staining Kit SDS PAGE (Catalog # 35076.01 SERVA, Heidelberg, Germany) (Figure S1).

#### 2.2.2. In-Solution Digestion

Proteins were precipitated using three times the volume of chilled acetone. Samples were incubated at −80 °C for 30 min, then at −20 °C overnight, and centrifuged at 10,000 RPM for 30 min [11]. The precipitated protein pellets were dissolved in 8 M urea (500 mM Tris, pH 8.5). Samples were quantified by Bradford assay using Pierce Detergent Compatible Bradford Assay Kit (Catalog # 23246, Thermo Fisher Scientific, Waltham, MA, USA). A mass of 40 micrograms of depleted plasma samples and albumin fractions were subjected to digestion. Samples were reduced using 2 µL of 200 mM dithiothreitol (DTT), followed by alkylation with 2 µL of 1 M iodoacetamide (IAA). Trypsin digestion was performed overnight at 37 °C at a ratio of 1:40, with samples diluted fourfold to reduce urea concentration and optimize trypsin activity [10].

#### 2.2.3. High-pH Reverse Phase Fractionation

Following digestion, peptides were desalted using the MonoSpin C^18^ column (GL Sciences, Tokyo, Japan) and fractionated using the high-pH reversed-phase fractionation method [12]. Seven pH buffers were used in this experiment, using two solutions: solution A: 5 mM Ammonium bicarbonate in MilliQ, and solution B: 5 mM ABC, 80% ACN. The pH was adjusted to 9 (basic pH), which allows the separation of the peptides based on their hydrophobicity. The high pH causes elution of peptide into fractions that are retained on the column under acidic conditions. In addition, the usage of 2 different solvents allows creating a spectrum of gradients to elute a wide diversity of peptides. The eluent linear gradient was achieved by increasing solution B with 6 different concentration fractions: 0%, 10%, 25%, 50%, 75% and 100% in solution A. Albumin fraction was then desalted on the same column and injected as fraction 7 using 100% solution B. Seven fractions were collected and subjected to speed vacuum prior to peptide quantification [13]. Resultant peptides were quantified by BCA assay using Pierce BCA Protein Assay Kit (Thermo Fisher Scientific, Waltham, MA, USA) [14].

#### 2.2.4. Liquid Chromatography–Tandem Mass Spectrometry (LC−MS/MS) Analysis

Peptides were analyzed by liquid chromatography–tandem mass spectrometry (LC−MS/MS) using an EASY-nanoLC 1200 system coupled with a Fusion Lumos Tribrid system (Thermo Fisher Scientific, Waltham, MA, USA). Peptides of concentration 200 ng (8 µL) were trapped on Thermo Scientific^TM^ Acclaim^TM^ (Glen Cove, NY, USA) PepMap^TM^ (Foster City, CA, USA) 100 (75 µm i.d, 2 cm long, 3 µm particles, C18) trap column. Peptides were then eluted on an Acclaim^TM^ PepMap^TM^ 100 analytical column with 75 µm i.d., 2 µm particles, 25 cm long, and C18 packing. LC gradient was performed using a 60 min gradient at flow rate 250 nL/min using solution (A); 0.1% formic acid and solution (B); 80% ACN and 0.2% formic acid and held for 4 min, followed by a 5 min ramp to 100% [15].

The mass spectrometer was utilized in data-dependent acquisition (DDA) mode, employing 2 s cycles for both the survey and MS/MS scans. Peptide precursor survey scans were conducted within the mass-to-charge ratio (m/z) range of 400 to 1800, with a resolution of 120,000. The automated gain control (AGC) and maximum ion injection time (IT) were set to auto-mode, following normal protocols. The process of selecting monoisotopic precursors (MIPS) was conducted at the peptide level, using an intensity threshold of 5 × 10^3^. Only peptides with charge states ranging from 2 to 7 were included for tandem MS analysis. The dynamic exclusion was set to 30 s with a 10 ppm mass tolerance, and isotopes were excluded. Isolation for MS2 scans was performed in the quadrupole with an isolation window of 1.5 m/z. The experimental procedure involved the implementation of higher-energy collisional dissociation (HCD) activation, with a collision energy (CE) of 30%, utilizing dynamic injection time mode and a 10,000 AGC target. The resulting fragments were detected using the rapid scan rate in the linear ion trap. MS1 and MS2 spectra were recorded in profile and centroid modes, respectively [15].

### 2.3. Untargeted Metabolomics Analysis

#### 2.3.1. Extraction of Metabolites from Plasma Samples

A volume of 50 µL of plasma was extracted using 400 μL of a precooled extraction solvent consisting of chloroform:water:methanol (1:1:3). The mixture was subjected to vortexing and agitation for at least 2 min, followed by ultrasonication for 15 min at 4 °C. Centrifugation of the solution was performed at a speed of 12,000 rpm at 4 °C for 10 min. The samples underwent drying using the Eppendorf concentrator plus at a temperature of 30 °C. The extract was rehydrated using a solvent composed of water, methanol, and acetonitrile at a ratio of 2:1:1, respectively, and afterwards subjected to LC-MS/MS analysis [11].

#### 2.3.2. Metabolome Profiling Using DDA-Based LC-MS/MS (Triple TOF-5600^+^)

Extracted metabolites were analyzed using LC–MS/MS analysis in both positive and negative ionization modes, utilizing a data-dependent acquisition (DDA) approach. Ultra-high-performance liquid chromatography (UHPLC) was performed on an ExionLC^TM^ (Framingham, MA, USA) AC UHPLC system (AB SCIEX, Concord, ON, Canada) coupled to a TripleTOF 5600+ mass spectrometer (AB SCIEX, Concord, ON, Canada). Chromatographic separation was achieved using an Acquity XSelect HSS T3 analytical column (2.1 × 150 mm, 2.5 µm; Waters Co., Milford, CT, USA). A total of 5 μL of each sample was injected and eluted over 28 min using a gradient elution program at a constant flow rate of 300 µL/min. The mobile phase solutions were as follows: mobile phase solution (A), 5 mM ammonium formate in 1% methanol (pH 3.0) for positive mode; solution (B), acetonitrile for both positive and negative modes; and solution (C), 5 mM ammonium format in 1% methanol (pH 8.0) for negative mode elution. Gradient elution was sustained at 0% B for 1 min, 0% B to 90% B in 20 min, 90% for 4 min, 90% B to 0% B in 0.1 min, and finally re-equilibrated with 0% B for 3 min [15].

Mass spectrometric analysis was conducted using a DuoSpray^TM^ ion source (AB SCIEX, Concord, ON, Canada) in both positive (ESI^+^) and negative (ESI^−^) ionization modes. In positive mode, the ions spray voltage capillary was set at 4500 eV, with a declustering potential of 80 V. In negative mode, the capillary voltage was −4500 eV, with a declustering potential of −80 V. The source temperature was maintained at 500 °C, with curtain gas pressure at 25 psi and both Gas 1 and Gas 2 pressures set at 45 psi. Collision energies were set to 35 V for positive mode and −35 V for negative mode, with a collision energy spread of 20 V. Data acquisition was collected based on DDA mode, selecting the 15 most intense ions while excluding isotopes within 2 Da using a mass tolerance of 10 ppm. MS/MS spectra were acquired at an ion selection threshold of 200 counts per second (cps). Data collection batches were managed using Analyst TF 1.7.1 software. To ensure data quality and stability, quality control (QC) samples were generated by pooling 5 μL from each sample and injecting every 10 samples throughout the sequence. Automatic mass calibration was performed every 2 h using an APCI calibration solution (AB SCIEX, Concord, ON, Canada).

### 2.4. Bioinformatics Analysis

#### 2.4.1. Shotgun Proteomics Data Analysis

Raw mass spectrometry data files were processed using Proteome Discoverer 1.4 (version 2.4.0.305, Thermo Scientific, Waltham, MA, USA) for peptide/protein identification. The search was performed against the human UniProt FASTA database (Swissprot canonical, 2023 version, containing 20,434 entries) using the Sequest HT search engine and a label-free quantification (LFQ) approach. Peptide searches allowed for fully and semi-tryptic peptides with up to two missed cleavages, with peptide lengths ranging from 6 to 144 amino acids. The precursor and fragment mass tolerances were set to 20 ppm and 0.5 Da, respectively. Fixed and variable modifications were assigned as follows. Carbamidomethylation of cysteine (+57.02146 amu) was set as a static modification. Variable modifications included oxidation of methionine (+15.995), acetylation of protein N-terminal and lysine (+42.01 amu), and pyrrolidone from carbamidomethylated C (−17.03 amu). To ensure high-confidence peptide and protein identification, a precolator was used for target/decoy validation, and results were filtered based on q-value estimation. A false discovery rate (FDR) of 1% was applied at both the peptide (minimum 6 amino acids) and protein levels.

#### 2.4.2. Metabolomics Data Analysis

For metabolite and small molecule identification, MSDAIL 4.0 was used [16]. The Human Metabolome Database (HMDB) version 4.0, containing 114,100 metabolites, was used as the search space (https://hmdb.ca). Fragment ions were filtered and stratified based on their relative intensity (SPLASH). Main fragments (base peaks) with a relative intensity >75% were included, while those ≤5% were excluded. Fragments with 5–75% intensity were classified as secondary fragments. PeakView 2.2 with MasterView 1.1 (AB SCIEX) was used to detect parent and fragment ions and manually validate data accuracy. A mass shift was applied to the alignment sheet exported from MS-DAIL for positive and negative ionization modes separately, with a precursor mass tolerance of 10 ppm. The sample’s signal was set at least five times higher than the blank [17]. The identified metabolites and small molecules were then subjected to statistical analysis and biological interpretation.

#### 2.4.3. Single-Omics Data Analysis

All statistical analysis and graphical representations were performed using R [18]. Protein quantification in this study was performed for ARMS/control, ERMS/control, and ARMS/ERMS comparisons. Protein abundances were calculated using the summed abundance parameter [19]. Unique and razor peptides were used for quantification, and precursor abundance was calculated based on intensity. Normalization was performed using the total peptide amount. Protein ratio calculations were conducted using a pairwise ratio based on a background-adjusted *t*-test. For metabolomics, data were normalized using Probabilistic Quotient Normalization (PQN) [20]. Filtration criteria were applied to both proteomics and metabolomics datasets, where proteins and metabolites identified in less than 50% and 80% of samples, respectively, in each group were excluded. Features that passed the filtration criteria underwent imputation using the conventional median imputation method [21,22], where missing values were replaced with randomized values within ±1% of the median of each group.

Both proteomics and metabolomics datasets were log-transformed and auto-scaled. A paired Student’s *t*-test was applied across the three groups to identify significantly expressed features. Multiple testing correction as performed using the false discovery rate (FDR), with an adjusted *p*-value threshold of <0.05. Features were further refined by applying an absolute log2 fold change (log2 FC) threshold of ≥1 to identify differentially expressed proteins and metabolites. Differentially expressed proteins (DEPs) and differentially expressed metabolites (DEMs) were subjected to hierarchical clustering analysis, volcano plot visualization, and Principal Component Analysis (PCA) using R.

For proteomics data, gene ontology (GO) and pathway enrichment analysis were conducted using Ensembl 92 and the Kyoto Encyclopedia of Genes and Genomes (KEGG) (Release 86.1) through ShinyGo V.0 [23]. For metabolomics data, pathway enrichment analysis was performed using MetaboAnalyst 5.0, with KEGG as a reference. DEMs were used as input to identify significantly enriched metabolic pathways, with a significance threshold of *q*-value < 0.05 [24,25].

#### 2.4.4. Weighted Gene/Metabolite Co-Expression Network Analysis

Weighted gene co-expression network analysis (WGCNA) and weighted metabolite co-expression network analysis (WMCNA) were performed using the WGCNA package in R [26]. To identify patterns of co-expressed genes or metabolites across samples. A similarity matrix was first constructed by calculating Pearson correlation coefficients between all pairs of genes or metabolites. This matrix was then transformed into a weighted adjacency matrix by applying a soft-thresholding power (power 8 for metabolomics and 10 for proteomics), selected to ensure scale-free network topology.

Subsequently, a topological overlap matrix (TOM) was computed to capture both direct and indirect interactions between nodes based on shared neighbors. A dissimilarity measure (1−TOM) was used for hierarchical clustering to identify modules of co-expressed molecules. Modules were detected using the cutTreeDynamic function with average linkage, mergeCutHeight = 0.25, and minModuleSize = 35 for proteomics and 20 for metabolomics. Modules positively correlated with RMS subtypes and showing an FDR-adjusted *p*-value < 0.05 were considered statistically significant.

For key driver analysis, protein–protein interaction (PPI) networks were constructed for each RMS subtype using the STRING database [27]. These networks incorporated upregulated DEPs, subtype-unique proteins, and hub proteins from significant WGCNA modules. Key driver proteins were identified based on centrality measures, including degree, betweenness, eigenvector, and PageRank centrality. Similarly, hub metabolites from significant WMCNA modules were examined to identify key metabolic interactions specific to each RMS subtype.

#### 2.4.5. Multi-Omics Integration Analysis

To comprehensively analyze the associations between the proteomics and metabolomics data, we applied Multi-Omics Factor Analysis (MOFA) and Data Integration Analysis for Biomarker Discovery using Latent Components (DIABLO). For MOFA, the data were scaled before analysis, and the model was trained using six latent factors with default training settings. This approach facilitated clustering, feature selection, and the exploration of cross-omics relationships [28].

Additionally, DIABLO from the mixOmics package was used to construct a correlation network between the proteomic and metabolomic datasets. A maximum of 25 features per component was selected, and pairwise similarities were computed using a Pearson similarity matrix. This enabled the identification of key biological pathways and molecular interactions associated with RMS subtypes [29]. The resulting networks were exported and visualized using Cytoscape 3.9.0 [30].

## 3. Results

### 3.1. Sample Cohort and Data Visualization of Rhabdomyosarcoma (RMS)

Our study included a cohort of 35 RMS patients, containing 18 ERMS and 17 ARMS cases, along with 18 healthy controls who met specific exclusion criteria. Among ERMS patients, 44.4% were male and 55.6% were female, 88.9% had tumors larger than 5 cm, and all had clinically involved lymph nodes. Additionally, 50% were classified as stage III, and 50% were post-surgical and categorized as group 3. Risk stratification was evenly distributed in ERMS, with 50% classified as high risk and 50% as intermediate risk (Table S1). Similarly, in the ARMS group, 41.2% were male and 58.8% were female, 83.3% had tumors larger than 5 cm, and 66.7% had clinically involved lymph nodes. Half of the ARMS patients were classified as stage III, while 44.4% were in group 3. Risk stratification in ARMS showed 52.9% high risk and 47.1% intermediate risk. The control group was sex-matched, comprising 77.8% males and 22.2% females, and showed no evidence of tumor characteristics or abnormal blood parameters.

### 3.2. Single-Omics Profiling Uncovered Possible Biomarkers Associated with Individual RMS Subtypes

**Figure 1:**
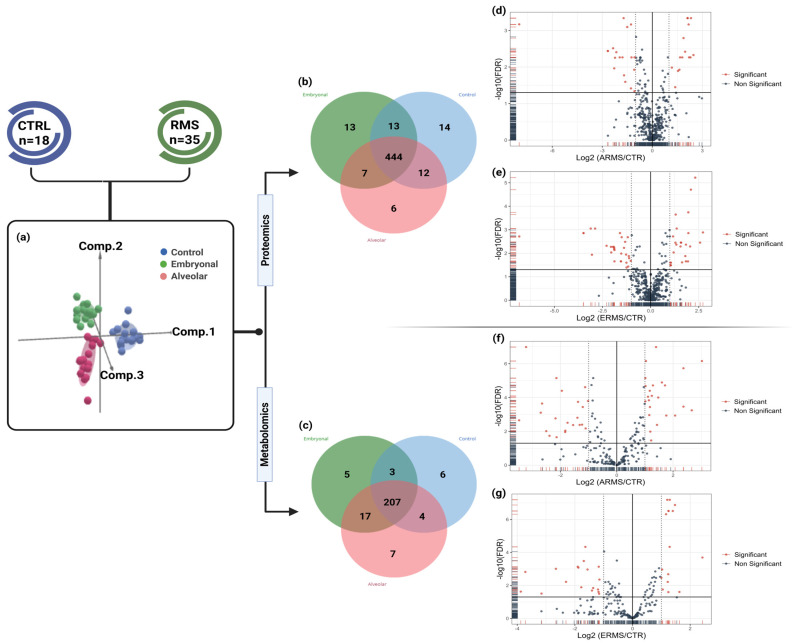
Comprehensive single-omics analysis of untargeted metabolomics and proteomics in RMS patients and controls. (**a**) A 3D scatter plot illustrating clustering of the three groups: embryonal RMS (green), alveolar RMS (red), and control (blue). (**b**) Venn diagram of identified proteins highlighting unique and shared proteins among ERMS, ARMS, and control groups. (**c**) Venn diagram of identified metabolites showing unique and shared metabolites across the three groups. (**d**) Volcano plot representing differentially expressed proteins (DEPs) between ERMS and control samples, with significant proteins indicated in red. (**e**) Volcano plot of DEPs between ARMS and control samples, highlighting significant proteins in red. (**f**) Volcano plot of differentially expressed metabolites (DEMs) between ERMS and control groups, with significant metabolites shown in red. (**g**) Volcano plot of DEMs between ARMS and control groups, with significant metabolites indicated in red.

Sample distribution analysis illustrated in 3D-scatter plots showed distinct clustering between RMS subtypes and control samples across the combined proteome and metabolome datasets (Figure 1a). Proteins and metabolites identified from single-omics were illustrated in a Venn diagram, showing both shared and unique molecules across the experimental groups (Figure 1b,c). As shown, the control group had 14 unique proteins not detected in RMS groups, including SPARC and thrombospondin-2, which regulate the extracellular matrix (ECM). The ARMS and ERMS subtypes had six and thirteen unique proteins, respectively. Interestingly, G1/S-specific cyclin-E1, and prolyl endopeptidase FAP were unique in ARMS, denoting its crucial role in cell cycle progression and tumor invasion. On the other hand, myosin-9, serum amyloid A-2, and Protein S100-A11 were reported only in ERMS. The latter proteins indicate alterations in muscle structure, inflammation, and tumor cell migration (Table S2). An observation markedly characterized the embryonal subtype. Collectively, all groups shared 444 proteins, which were subjected later to further analysis (Figure 1b).

In parallel, untargeted metabolomics analysis identified six unique metabolites in the control group, primarily associated with carbohydrate metabolism and phospholipid integrity (Table S2). The ARMS group identified seven distinct metabolites, among them tetrahydrobiopterin, linked to neurotransmitter synthesis and oxidative stress, deoxycholic acid, involved in lipid metabolism, and cadaverine, a polyamine that may contribute to tumor proliferation. In contrast, the ERMS group showed five unique metabolites. 1-methylnicotinamide was the most prominent, associated with NAD+ metabolism and cellular energy regulation, and other metabolites suggesting alterations in energy and lipid metabolism (Table S2). These findings suggest potential metabolic distinctions and subtype-specific adaptations in RMS. Furthermore, 207 metabolites were common and quantitatively evaluated among the groups (Figure 1c).

Differential expression analysis using volcano plots identified statistically significant proteins and metabolites across experimental groups (Figure 1d–g). In the ARMS subtype, 35 DEPs were detected, including 16 upregulated and 19 downregulated (*q* < 0.05, |log_2_FC| ≥ 1) (Figure 1d). The ERMS group showed 57 DEPs, with 27 upregulated and 30 downregulated relative to controls under the same threshold (Figure 1e). Metabolomic analysis revealed 46 DEMs in ARMS (24 upregulated, 22 downregulated; *q* < 0.05, |log_2_FC| ≥ 1) and 34 DEMs in ERMS (16 upregulated, 18 downregulated; *q* < 0.05, |log_2_FC| ≥ 1) (Figure 1f–g). These significant features, integrated with clinical data, were visualized using hierarchical clustering heatmaps (Figure S2a,b), illustrating distinct molecular expression patterns across the subtypes.

### 3.3. Gene Ontology (GO) and Pathway Enrichment Analysis of DEPs and DEMs

To explore the functional implications of molecular alterations in RMS, enrichment analyses were performed on both proteomics and metabolomics datasets. DEPs and DEMs, including unique molecules in each subtype, were included and analyzed to identify their associated biological processes and pathways (Tables S2 and S3).

**Figure 2:**
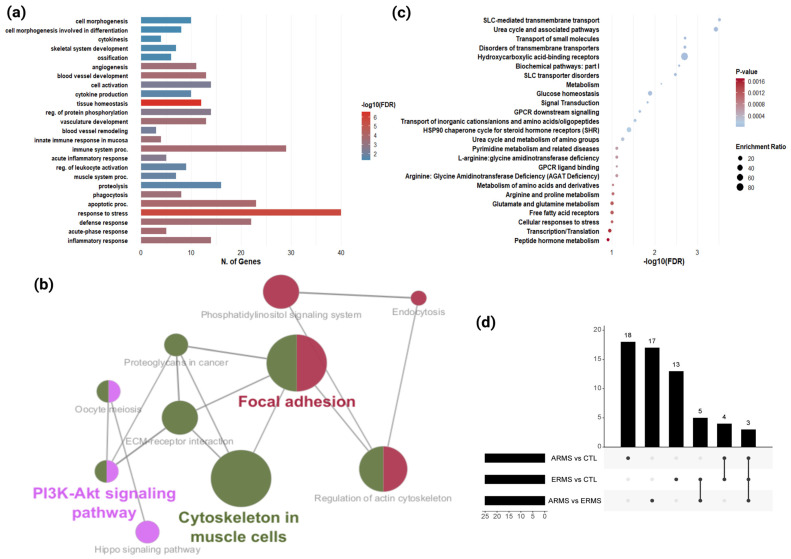
Descriptive and enrichment analysis for identified proteins and metabolites. (**a**) Bar plot of enriched biological processes derived from merged data of DEPs and unique proteins identified in ERMS/control and ARMS/control comparisons. (**b**) Network plot of significantly enriched pathways identified using KEGG database for merged profile of ERMS/control and ARMS/control comparisons with FDR < 0.05. (**c**) Top 25 significant pathway enrichment analysis using RaMP-DB with FDR < 0.05 for merged metabolite list from ERMS/control and ARMS/control comparisons. (**d**) UpSet plot showing significant pathways identified from each comparison (ARMS/Control, ERMS/Control, and ARMS/ERMS) and illustrating intersections and unique overlaps.

GO enrichment analysis of DEPs identified key biological processes associated with cell morphogenesis, skeletal system development, and ossification (Table S5). Involved proteins such as collagen type I alpha 1 (COL1A1), serglycin (SRGN), lactotransferrin (LTF), cartilage oligomeric matrix protein (COMP), and calreticulin (CALR), might be critical for RMS differentiation (Figure 2a). Tumor-associated angiogenic pathways, including blood vessel development and vascular remodeling were also enriched with major contribution of myosin-9 (MYH9), endoglin (ENG), thrombospondin-1 (THBS1), fibronectin (FN1), platelet factor 4 (PF4), phospholipase D (PLD1), filamin A (FLNA), and angiotensin-converting enzyme (ACE). Notably, immune-related pathways also showed significant enrichment, indicating possible modulation of the tumor immune microenvironment. Many DEPs within this category such as neutrophil defensin 1 (DEFA1), neutrophil defensin 3 (DEFA3), PF4, actin cytoplasmic 1 (ACTB), THBS1, fibrinogen-like protein 1 (FGL1), and various immunoglobulins were downregulated compared to control, suggesting a potential immune suppressive state or impaired immune activation in RMS (Figure 2a, Tables S3 and S4). For each subtype (ERMS and ARMS), separate GO biological enrichment analyses were performed (Figure S2c,d).

KEGG pathway analysis further highlighted dysregulated signaling and adhesion pathways in RMS. The PI3K-Akt signaling and proteoglycans in cancer were significantly enriched, involving proteins such as COL1A1, FN1, THBS1, FLNA, and ACTB (Figure 2b). Additional enrichment was observed in ECM-receptor interaction, focal adhesion, and actin cytoskeleton dynamics, driven by proteins including MYH9, profilin-1 (PFN1), and thymosin beta-4 (TMSB4X). The Hippo signaling pathway, which regulates proliferation and differentiation, was also enriched with involvement of 14-3-3 proteins (YWHAB, YWHAZ) and ACTB (Table S6). To delineate pathway contributions specific to each RMS subtype, separate KEGG enrichment analyses were also explored using DEPs and unique proteins of ERMS and ARMS (Figure S2e,f).

Pathway enrichment analysis of differentially expressed and unique metabolites using the curated RaMP-DB database unleashed several metabolic perturbations relevant to RMS biology (Figure 2c, Table S7). Among the most significantly enriched pathways (*q* < 0.05), SLC-mediated transmembrane transport (enrichment ratio = 7.6, *q* = 0.0003), transport of small molecules (enrichment ratio = 5.7, *q* = 0.0020), and disorders of transmembrane transporters (enrichment ratio = 8.7, *q* = 0.0020) were prominently overrepresented. These pathways were mainly driven by key metabolites such as proline, lactic acid, oleic acid, and 3-hydroxybutyric acid, suggesting potential impaired metabolite exchange and trafficking across cellular membranes. Additionally, the urea cycle and associated amino acid metabolism pathway were significantly enriched (enrichment ratio = 21.4, *q* = 0.0004), suggesting a possible disruption in nitrogen disposal and amino acid biosynthesis. Contributing metabolites included ornithine, L-aspartic acid, and N-acetyl-L-glutamic acid.

Several metabolites contributed to pathways involved in lipid and energy metabolism showed a significant enrichment score. Notably, 3-hydroxybutyric acid, cortisol, and glycocholic acid were associated with enrichment of hydroxycarboxylic acid-binding receptors (enrichment ratio = 80.2, *q* = 0.002), glucose homeostasis (enrichment ratio = 20.4, *q* = 0.013), and broader metabolism-related pathways (enrichment ratio = 3.6, *q* = 0.007). In addition, multiple metabolites mapped to signaling-related pathways, including G protein-coupled receptors (GPCR) downstream signaling (enrichment ratio = 5.1, *q* = 0.022) and signal transduction (enrichment ratio = 3.7, *q* = 0.015), indicating potential metabolic crosstalk with oncogenic signaling processes. Key contributors to these pathways included progesterone, sphingosine-1-phosphate, and nicotinic acid (Figure 2c, Table S7).

To further dissect subtype-specific metabolic alterations, enrichment analyses were performed independently for DEMs and unique metabolites from ERMS and ARMS (Figure S2g,h). An UpSet plot summarizes the shared and distinct enriched pathways across subtypes, highlighting both common metabolic features and unique signatures (Figure 2d, Table S7).

### 3.4. Subtype-Specific Differentiation of Rhabdomyosarcoma by Weighted Gene and Metabolite Co-Expression Network Analysis (WGCNA/WMCNA)

To identify robust molecular signatures reflective of RMS phenotypic traits based on the metagene concept (clusters of co-expressed genes or metabolites that act as unified markers), we performed WGCNA on proteomics data and WMCNA on metabolomics data.

**Figure 3:**
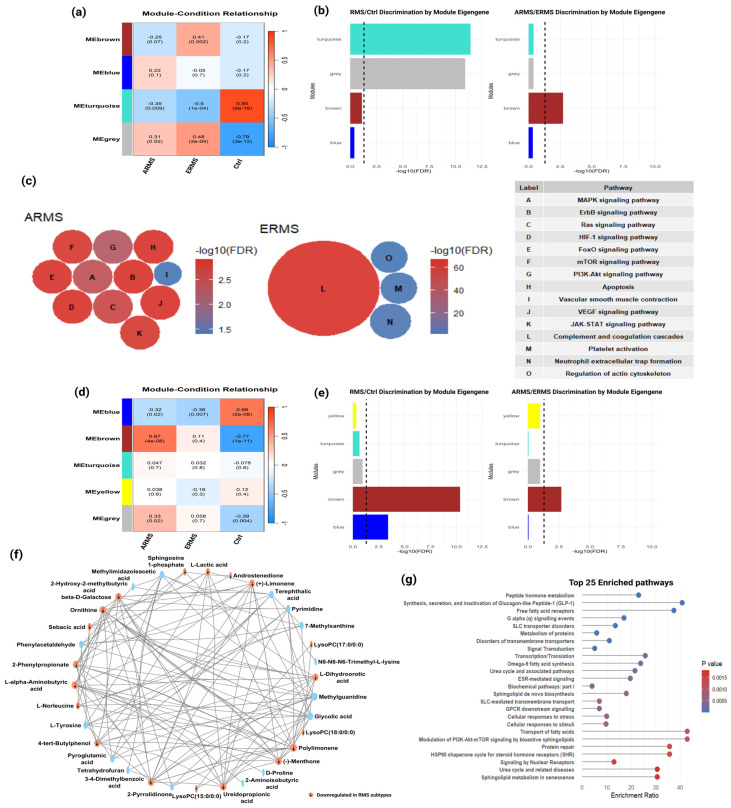
Co-expression network of identified proteins and metabolites constructed by weighted gene/metabolite co-expression network analysis. (**a**) Heatmap representing correlation of module eigengenes with phenotypes, illustrating relationships between modules and conditions: normal control (right), ERMS (middle), and ARMS (left). (**b**) Bar plot depicting module discrimination based on module eigengenes, highlighting the turquoise module’s strong correlation with control samples, the brown module’s strong correlation with ERMS, and the blue module’s strong correlation with ARMS. (**c**) Bubble chart representing pathway enrichment for the brown (ERMS) and blue (ARMS) modules; bubble color indicates FDR (−log10), bubble size reflects number of genes annotated in each pathway, and letters correspond to pathways listed in the accompanying table. (**d**) Heatmap illustrating correlation of module eigenmetabolites with groups, displaying relationship between modules and conditions: normal control (right), embryonal RMS (middle), and alveolar RMS (left). (**e**) Bar plot showing module discrimination by module eigenmetabolites, emphasizing the blue module’s strong correlation with control samples and the brown module’s correlation with ARMS. (**f**) Network analysis of metabolites identified in the blue module, which is highly correlated with control samples and the arrows shows downregulated metabolites in RMS subgroups. (**g**) Pathway enrichment analysis of metabolites identified in the brown module (ARMS).

WGCNA of the proteomics dataset identified four distinct co-expression modules, each of which demonstrated unique functional associations (Figure S3a,b, Table S8). Module sizes ranged from 86 proteins (turquoise) to 218 proteins (grey). Correlation analysis showed significant associations between these modules and experimental groups (Figure 3a,b). Notably, the turquoise module exhibited a strong positive correlation with the control group (*r* = 0.85, *p* = 9 × 10^−16^), indicating that proteins within this module were predominantly downregulated in RMS subtypes or uniquely present in the control group (Figure S3c, highlighted in orange). These proteins were primarily involved in immune regulation, structural integrity, and cytoskeletal organization (Table S9), suggesting their relevance in maintaining normal tissue function. Conversely, the brown module displayed a significant positive correlation with ERMS (*r* = 0.41, *p* = 0.002), while the blue module showed a modest association with ARMS (*r* = 0.22, *p* = 0.1) (Figure 3a,b). These modules were enriched with proteins that were either upregulated or unique to their respective subtypes, an observation that proves the concept of subtype-specific molecular signature patterns.

To further investigate the biological functions of these RMS-associated modules, we conducted KEGG pathway enrichment analysis. The brown module, associated with ERMS, was significantly enriched for pathways related to complement and coagulation cascades, platelet activation, neutrophil extracellular trap formation, and regulation of the actin cytoskeleton (Figure 3c). This module included numerous key effectors such as complement proteins (C1QA, C1QB, C3, C4A, etc.), coagulation factors (F2, F5, F10, etc.), and extracellular matrix proteins, proposing potential roles in immune modulation and hemostasis (*n* = 36, *q* < 0.05) (Figure S3d, Table S10). In contrast, the blue module, associated with ARMS, despite being highly populated by immunoglobulin-related proteins, was functionally enriched in oncogenic signaling pathways, including PI3K-Akt, MAPK, mTOR, ErbB, Ras, VEGF, JAK-STAT, and HIF-1 pathways (Figure 3c). Interestingly, this enrichment was specifically driven by AKT1, AKT2, and CALD1, which were the only proteins within the blue module that mapped to these signaling cascades.

To better understand the regulatory mechanisms within these modules, we constructed protein–protein interaction (PPI) networks that identified key hub proteins (Figure S3e,f, Table S11). These networks revealed strong interactions among hub proteins, DEPs, and proteins unique to each RMS subtype, supporting their central roles in driving the co-expression patterns and molecular differences observed in RMS [31].

To further characterize metabolic alterations in RMS, we performed WMCNA, which identified five metabolite modules with distinct co-expression patterns (Figure 3d,e). Correlation analysis revealed significant associations between specific modules and RMS subtypes (Figure S3g,h, and Table S12). The blue module showed a strong positive correlation with the control group (*r*  =  0.68, *p*  =  2 × 10^−8^), while the brown module was significantly associated with ARMS (*r*  =  0.67, *p*  =  4 × 10^−8^). No module showed a significant association with ERMS (Figure 3d).

Metabolites within the control-associated blue module included several amino acids, including L-tyrosine, L-α-aminobutyric acid, ornithine, methionine, D-proline, and DL-2-aminooctanoic acid, along with bioactive lipids such as sphingosine-1-phosphate, deoxycholic acid glycine conjugate, and lysophosphatidylcholines (LysoPCs) (Figure 3f). The marked absence or downregulation of these metabolites in both ARMS and ERMS suggests subtype-specific disruptions in amino acid metabolism, protein turnover, and lipid-mediated signaling in RMS.

Pathway enrichment analysis of the ARMS-associated brown metabolite module highlighted alterations in hormone regulation, lipid signaling, and membrane transport. Peptide hormone metabolism was the most enriched pathway (*q* = 0.00043), reflecting changes in endocrine regulation. The GLP-1 pathway, critical for glucose and insulin homeostasis, was also significantly enriched (*q* = 0.00218). Additionally, lipid signaling pathways, including free fatty acid receptors and G alpha (q) signaling, showed marked enrichment (*q* ≤ 0.0051). A possible shift in metabolic plasticity linked to tumor growth might be the case here in ARMS. Notably, enrichment of SLC transporter dysfunction (*q* = 0.0133) suggests impaired membrane transport affecting nutrient exchange. Further enrichment in protein metabolism and signal transduction pathways (*q* ≤ 0.0291) supports a broad systemic metabolic reprogramming in ARMS. Collectively, these results define a distinctive metabolic profile associated with ARMS (Figure 3g; Table S13).

### 3.5. Comprehensive Multi-Omics Integration of Untargeted Proteomics and Metabolomics

**Figure 4:**
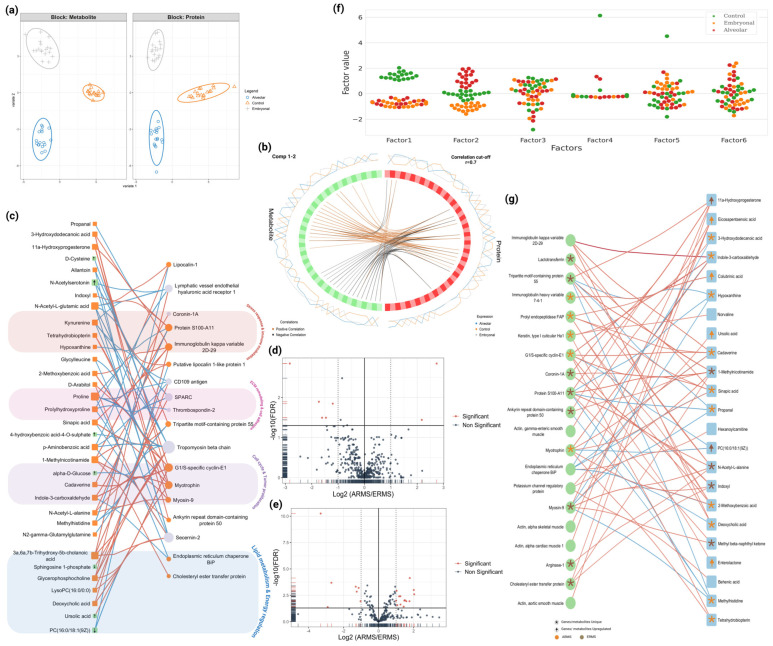
Integrative multi-omics analysis of untargeted proteins and metabolites data. (**a**) Sample plot of DIABLO model illustrating separation between ERMS (grey), ARMS (blue), and control (orange) groups. (**b**) Circos plot showing correlated metabolites (green) and proteins (red) selected by DIABLO, with lines representing significant correlations (|r| ≥ 0.7): orange for positive, black for negative. Outer line plots show expression across groups (blue: alveolar, orange: control, gray: embryonal). “Comp 1-2” indicates first two DIABLO components used for integration. (**c**) DIABLO network analysis of first component, containing 18 proteins (grey circles) and 33 metabolites (green squares). Unique proteins and metabolites identified from single-omics are highlighted in orange; arrows indicate upregulation. Negative correlation edges (49) are marked in red, while positive correlation edges (53) are in blue. (**d**) Volcano plot of differentially expressed proteins (DEPs) between ARMS and ERMS. (**e**) Volcano plot of differentially expressed metabolites (DEMs) between ARMS and ERMS. (**f**) Score plots for six latent factors (LFs) in MOFA. The y-axis represents LF scores for each sample, with colors representing sample groups: green (control), orange (ERMS), and red (ARMS). (**g**) MOFA network analysis for factor two, containing 20 proteins (green circles) and 23 metabolites (blue squares). Unique proteins and metabolites identified from single-omics are marked with (*); arrows indicate upregulation (orange for ARMS and brown for ERMS). Negative correlation edges (18) are highlighted in red, while positive correlation edges (57) are in blue.

To gain deeper insights into RMS and elucidate the molecular interplay between metabolites and proteins, we performed a comprehensive multi-omics integration using both supervised (DIABLO) and unsupervised (MOFA) approaches. This integration was conducted using the same proteomics and metabolomics datasets described earlier to ensure consistency across analyses. A statistical correlation network between the different omics analyzed was constructed to identify significant co-regulation patterns while minimizing biases associated with knowledge-based frameworks (Figure S4a,b). DIABLO analysis effectively distinguished control samples from RMS subtypes, with clear separation observed in the component space (Figure 4a). Interestingly, component 1 separated control samples from RMS, while component 2 further differentiated ARMS from ERMS (Figure S4c,d). These findings emphasize the distinct molecular signatures between RMS subtypes and controls.

The correlation network derived from component 1 revealed 49 negative correlation edges (median r = −0.731) and 53 positive correlation edges (median r = 0.76), representing interactions between 18 proteins and 33 metabolites (Figure 4b and Table S14). These correlations incorporate both differentially regulated and subtype-unique molecules, significantly expanding the earlier findings reported in single-omics analyses. Pathway enrichment analysis of the correlated entities closely aligned with the results from WGCNA and WMCNA (Table S15), reinforcing the biological relevance of the integrated network. The four major functional categories that emerged were stress response and immune modulation, ECM remodeling and cell adhesion, cell cycle and tumor proliferation, and lipid metabolism and energy regulation (Figure 4c). This overlap demonstrates that multi-omics integration not only validates single-omics discoveries but also provides a comprehensive new paradigm of coordinated molecular regulation across the proteome and metabolome, offering a more holistic view of RMS biology.

To further validate and expand the integrative insights from DIABLO, we applied MOFA as an unsupervised multi-omics factor analysis framework using the same set of proteins and metabolites. MOFA independently captured the major sources of variance and clearly distinguished between RMS subtypes and controls. Consistent with single-omics analyses (Figure 4d,e), MOFA identified subtype-specific molecular signatures, with Factor 1 (latent factor 1) primarily separating RMS from controls, and Factor 2 differentiating ARMS from ERMS (Figure 4f). Both the proteomics and metabolomics datasets contributed strongly to these latent factors (Figure S4b), and the robustness of the model was supported by clear group separation and strong cross-validation metrics (Figure S4e,f).

Notably, Factor 2 revealed 75 significant protein–metabolite correlations, comprising 57 positive and 18 negative associations between 20 proteins and 23 metabolites (Figure 4g). These correlated features exhibited strong overlap with DIABLO’s discriminative component 2 and included shared DEPs, DEMs, and subtype-unique molecules (Figure S4g–j, Table S16), reinforcing the robustness of the integrative approach.

In ARMS, several proteins were uniquely expressed. For instance, G1/S-specific cyclin-E1, myotrophin, immunoglobulin kappa variable 2D-29, prolyl endopeptidase FAP, keratin type I cuticular Ha1, and immunoglobulin heavy variable 7-4-1 exhibited strong positive correlations with ARMS-specific metabolites such as indole-3-carboxaldehyde (r up to 0.91), cadaverine, 2-methoxybenzoic acid, sinapic acid, and tetrahydrobiopterin. Cyclin-E1 and myotrophin were positively correlated with indole-3-carboxaldehyde (r = 0.91 and 0.86, respectively) and tetrahydrobiopterin (r = 0.74 and 0.73, respectively) (Figure 4g). Conversely, PC(16:0/18:1(9Z)), a metabolite significantly downregulated in ARMS, showed negative correlations with ARMS-unique proteins (cyclin-E1, r = −0.71; myotrophin, r = −0.68), which may reflect subtype-specific alterations in lipid metabolism related to tumor progression (Figure 4g). Notably, endoplasmic reticulum chaperone BiP, a control-specific protein, showed negative correlations with several ARMS-related metabolites (e.g., r = −0.74 with indole-3-carboxaldehyde), indicating altered relationships in ARMS compared to controls.

In ERMS, MOFA Factor 2 revealed strong positive correlations between ERMS-unique proteins, including S100-A11, myosin-9, Tripartite motif-containing protein 55 (TRIM55), ankyrin repeat domain-containing protein 50, cholesteryl ester transfer protein, coronin-1A, lactotransferrin, and arginase-1, and ERMS-enriched metabolites such as 1-methylnicotinamide (r up to 0.89), 11α-hydroxyprogesterone, N-acetyl-L-alanine, indoxyl, and methyl β-naphthyl ketone. These associations point to immune-modulatory and muscle differentiation programs specific to ERMS. Interestingly, control-specific metabolites, including methylhistidine, hypoxanthine, and propanal, were negatively correlated with ERMS proteins (e.g., S100-A11 and TRIM55, r~−0.77), indicating repression of homeostatic pathways (Figure 4g).

Additionally, MOFA uncovered biologically relevant protein–metabolite interactions that were not highlighted by single-omics analyses. For example, actin isoforms from cardiac, skeletal, and smooth muscle types emerged as central hubs, showed strong correlation with eicosapentaenoic acid (r~0.74) (Figure 4g). This association suggests a potential link between cytoskeletal remodeling and lipid-mediated signaling in RMS, reflecting coordinated regulation of structural integrity and metabolic adaptation.

To further explore the functional relevance of subtype-specific features, we conducted GO enrichment analysis on the proteins associated with MOFA Factor 2 (which distinguishes ARMS from ERMS) and overlapping with DIABLO’s discriminant features. The analysis revealed significant enrichment in biological processes specific to muscle differentiation, striated muscle contraction, and bone maturation (Figure S4k). These findings indicate that the key proteins driving molecular separation of RMS subtypes are involved in developmental and differentiation pathways, supporting their potential role in RMS pathogenesis. Integrating these protein features with their correlated metabolites enhanced biological interpretability and emphasized the importance of exploring coordinated protein–metabolite relationships in RMS subtype stratification.

The consistent stratification of RMS subtypes by both supervised (DIABLO) and unsupervised (MOFA) approaches highlights the robustness of the multi-omics integration strategy and affirms the distinct, coordinated molecular programs underlying ARMS and ERMS pathophysiology.

### 3.6. Association of Clinical Characteristics with Proteomic and Metabolomic Profiles

To explore molecular patterns associated with clinical parameters in RMS, we performed Orthogonal Partial Least Squares Discriminant Analysis (OPLS-DA) on proteomic and metabolomics datasets. Our data revealed a significant association with risk stratification, lymph node involvement, and age (Figure S5). For risk stratification, high-risk patients exhibited elevated clusterin (VIP = 2.98) and keratin-6A (VIP = 2.43), indicating stress response and cytoskeletal remodeling, while AKT isoforms (VIP = 2.07) and immunoglobulin chains (VIP = 2.05–2.53) were enriched in intermediate-risk patients (Figure S5a,b). Metabolomics analysis highlighted increases in glutaconic acid (VIP = 3.08), *p*-hydroxymandelic acid (VIP = 2.51), and methionine (VIP = 2.34) in intermediate- and high-risk patients (Figure S5c,d).

Not surprisingly, lymph node involvement seems to impact both proteomics and metabolomics profiles as well. Protein S100-A8 (Calgranulin-A) (VIP = 3.08) was elevated in patients without clinical lymph node involvement, while cysteine-rich secretory protein 3 (CRISP-3) (VIP = 2.97) was reduced, suggesting inflammation and metabolic adaptations (Figure S5e,f). Metabolomics identified undecanedioic acid (VIP = 3.04) and glyoxylic acid (VIP = 2.60) as key markers reflecting lipid and carbohydrate metabolism shifts (Figure S5g,h). For age stratification, younger patients (5–8 years) showed elevated biotinidase (VIP = 3.04) and apolipoprotein A-I (VIP = 2.82), while older children (>8 years) exhibited higher plasminogen-like protein B (VIP = 2.47) (Figure S5i,j), and homovanillic acid (VIP = 2.88), reflecting developmental differences in immune and metabolic pathways (Figure S5k,l). Collectively, these findings demonstrate that proteomics and metabolomics profiling may provide additional clinical relevance in understanding RMS pathophysiology and stratifying patients for precision approaches (Figure S5, Table S17).

## 4. Discussion

This study presents a comprehensive integrative multi-omics analysis of RMS, uncovering distinct molecular signatures and biological pathways that differentiate between its subtypes. By combining untargeted proteomics and metabolomics data, we identified DEPs and DEMs that distinguish ERMS and ARMS from healthy control samples. The application of network-based methods, including WGCNA and WMCNA, enabled the discovery of co-expression modules and key hub molecules involved in RMS pathogenesis. Furthermore, integrative frameworks such as MOFA and DIABLO provided complementary insights into subtype-specific molecular programs, reinforced and extended the findings observed from single-omics analyses.

In both omics layers, we identified differentially expressed proteins and metabolites that reflect hallmark processes of RMS. The GO and KEGG enrichment analyses of DEPs revealed key molecular processes underlying RMS pathogenesis, particularly those related to cellular differentiation, angiogenesis, immune modulation, and tumor progression. The enrichment of biological processes such as cell morphogenesis, skeletal system development, and ossification underscores the aberrant reactivation of developmental pathways in RMS. Proteins such as collagen type I alpha 1 (COL1A1), serglycin (SRGN), lactotransferrin (LTF), cartilage oligomeric matrix protein (COMP), and calreticulin (CALR), all implicated in extracellular matrix organization and bone/cartilage formation, are likely reflecting the mesenchymal origin and impaired differentiation state of RMS cells invasion [32,33]. These proteins may serve as markers of tumor differentiation status and have potential implications for disease stratification [34].

Both ERMS and ARMS subtypes exhibited downregulation of cadherin-5 (CDH5), a key endothelial adhesion molecule, implicating potential disruptions in vascular integrity and enhanced migratory capacity [35]. Given the reported regulatory effects of CALR on cadherin angiogenesis [36], our findings suggest a possible CALR–cadherin axis influencing tumor progression in RMS. Further investigation into CALR expression and its epigenetic impact on adhesion molecules may provide novel insights into the metastatic mechanisms of RMS. Although COL1A1 and SRGN have been reported as markers in several cancers, with their well-known implication in tumorigenesis, there is currently no evidence of their roles in RMS subtypes [37,38]. Interestingly, despite prior studies reporting elevated COMP expression in various cancers [39], our results revealed a significant downregulation of COMP in embryonal rhabdomyosarcoma. This divergence may reflect unique ECM remodeling dynamics or tumor–stroma interactions characteristic of ERMS.

Angiogenesis emerged as a central tumor-supportive process, with enriched pathways related to blood vessel development and vascular remodeling. In this study, such proteins were orchestrating endothelial cell migration and vessel stabilization, and all are crucial for neovascularization that sustains tumor growth. One such protein was endoglin (ENG). An earlier study reported upregulation of ENG in ARMS, proposing it as a candidate prognostic biomarker associated with decreased survival rates [40]. It is a pertinent angiogenesis marker expressed in various tumor types, particularly those located in the head and neck region [41]. Similarly, the overexpression of fibronectin (FN1) in ERMS observed in our study aligns with previous findings, which suggests that FN1 contributes to the stroma-rich appearance of some ERMS tumors [42].

However, the downregulation of key angiogenic inhibitors such as thrombospondin-1 (THBS1) and platelet factor 4 (PF4) suggests a disrupted regulatory balance that may promote unchecked neovascularization within the tumor microenvironment. THBS1 inhibits angiogenesis by inducing endothelial cell apoptosis, suppressing cell migration, and proliferation [43]. Similar function of downregulated PF4, known to inhibit endothelial cell migration and proliferation [44]. Furthermore, the downregulation of profilin-1 (Pfn1), a protein involved in actin polymerization, may contribute to tumor progression. Pfn1 loss has been associated with increased cancer cell proliferation, migration, and invasion across multiple tumor types [45].

The immune-related enriched molecules observed in this study indicate that RMS may engage in active modulation of the immune microenvironment. At the same time, downregulation of innate immune effectors, including neutrophil defensins (DEFA1/3), and fibrinogen-like protein 1 (FGL1), along with various immunoglobulin chains, might be the cause of impaired antigen presentation and immune surveillance in rhabdomyosarcoma. It might imply a shift toward an immunosuppressive tumor microenvironment, possibly favoring tumor progression by facilitating tumor immune evasion and progression in RMS, an observation reported in an earlier study [46]. It is worth mentioning that FGL1 has been reported to be downregulated in pancreatic, breast, liver, and head and neck cancers, which could be similar to our study due to the location of the rhabdomyosarcoma selected cases [47]. This aligns with previous reports highlighting immune evasion in pediatric soft tissue sarcomas [48].

The metabolic enrichment analysis reinforces the concept that RMS undergoes profound metabolic reprogramming to sustain its proliferative and survival advantages. Both ERMS and ARMS subtypes exhibited distinct alterations in pathways related to nutrient acquisition, biosynthesis, and energy metabolism, which are hallmarks of cancer metabolism [49]. A prominent alteration in solute carrier (SLC)-mediated transmembrane transport, regulating the uptake and export of amino acids, ions, and lipids essential for tumor metabolic flexibility, was reported in this study. Dysregulation of SLC activity may compromise nutrient availability or disrupt redox balance. These mechanisms have been implicated in different cancers, including sarcoma [50].

Disrupted nutrient and metabolite exchange across cellular compartments was largely driven by alterations in metabolites, including lactic acid, oleic acid, and ornithine. The significant downregulation of lactic acid, especially in ARMS, contrasts with the classic Warburg effect, where lactate accumulation is a byproduct of aerobic glycolysis [51]. This suggests a possible metabolic shift in ARMS, potentially favoring oxidative phosphorylation, increased lactate clearance, or altered glycolytic flux. Functionally, lower lactate levels could reduce extracellular acidification, thereby affecting ECM remodeling, invasion, and immune evasion. This may modulate tumor–stroma interactions via integrin signaling pathways such as PI3K-Akt [52]. The enrichment of the PI3K-Akt pathway in our proteomics data supports this connection. This finding aligns with the proteomic evidence for altered extracellular matrix and immune interactions, suggesting a tight interplay between metabolic state and tumor microenvironmental dynamics.

Ornithine downregulation in RMS, particularly in ARMS, adds further insight into nitrogen metabolism dysregulation. Ornithine, a urea cycle intermediate and precursor for polyamine biosynthesis, plays key roles in cell proliferation and differentiation. Its depletion may reflect enhanced polyamine synthesis to support cell proliferation, a phenomenon observed in several cancers, including hepatocellular carcinoma (HCC) [53]. Additionally, impaired ornithine availability may disrupt urea cycle function, contributing to nitrogen imbalance and cellular stress. Perturbations in nitrogen metabolism were further indicated by differential regulation of L-aspartic acid and N-acetyl-L-glutamic acid (NAG), both involved in amino acid biosynthesis and ureagenesis. The unique appearance of NAG in both ERMS and ARMS may reflect enhanced nitrogen disposal through the urea cycle upregulation, possibly compensating for increased protein catabolism and ammonia production in rapidly proliferating tumor cells [54].

Lipid metabolism also emerged as a critical node of metabolic reprogramming in RMS [55]. The shared upregulation of 3-hydroxybutyric acid and myristic acid in both RMS subtypes suggests increased ketone body metabolism and fatty acid turnover, which may serve as alternative energy sources for tumor cells under metabolic stress. This aligns with previous studies suggesting that RMS cells, particularly in nutrient-depleted environments, rely on lipid metabolism for survival and proliferation [55,56]. Targeting lipid metabolism has been shown to exert antitumor effects in RMS models, underscoring its therapeutic relevance.

Although the importance of fatty acid metabolism in RMS has been recognized, mechanistic studies focusing on fatty acid and bile acid pathways in RMS remain limited. To date, no detailed investigations have explored the functional roles of these metabolites in RMS pathogenesis, despite their known involvement in other cancers [57,58]. Interestingly, ERMS exhibited specific reductions in oleic acid and glycocholic acid, suggesting possible alterations in fatty acid metabolism and bile acid conjugation. While the biological implications of these changes in RMS are still unclear, they may reflect subtype-specific metabolic adaptations. Conversely, ARMS showed elevated levels of deoxycholic acid, a secondary bile acid known to promote inflammation and tumor progression in other malignancies [59]. These differences further underscore the metabolic heterogeneity between RMS subtypes. Notably, expression of fatty acid synthase (FASN), a key enzyme in de novo lipogenesis, has been proposed as a prognostic marker in soft tissue sarcomas, further supporting the relevance of fatty acid metabolism in sarcoma progression and potential therapeutic targeting [60].

Beyond nutrient metabolism, the involvement of metabolites such as cortisol, sphingosine-1-phosphate (S1P), and progesterone highlights the complex interplay between metabolic reprogramming and oncogenic signaling in RMS. S1P, a bioactive lipid, acts by binding to GPCRs on the cell surface and has been shown to play important roles in RMS by promoting angiogenesis, tumor cell proliferation, and metastasis [61]. Although S1P regulates cell survival and motility, its effects are context-dependent; some studies report that S1P promotes cancer cell proliferation, while others demonstrate antitumor effects through growth inhibition and apoptosis induction [62,63]. In our study, the consistent downregulation of S1P in both ERMS and ARMS may reflect suppressed sphingolipid biosynthesis, increased degradation, or a shift away from S1P receptor-mediated signaling, potentially altering angiogenic and immune-regulatory pathways [64]. Additionally, the detection of stress and steroid-related metabolites like cortisol suggests an adaptive tumor response to microenvironmental stressors [65]. While studies on cortisol’s role in sarcomas are limited, existing evidence indicates that cortisol can stimulate proliferation in various cancer cell lines, including rhabdomyosarcoma cells, supporting our findings [66]. Together, these findings emphasize the importance of hormone and lipid signaling within the tumor microenvironment and highlight potential therapeutic targets in RMS that warrant further investigations.

Network-based analyses via WGCNA and WMCNA further elucidated the molecular compartmentalization of RMS subtypes. In ERMS, the brown module showed significant enrichment of proteins involved in complement and coagulation cascades, suggesting that this subtype may actively modulate the tumor microenvironment through immune-related mechanisms and thrombotic mechanisms. Although previous studies have linked these pathways to RMS broadly [67], our analysis highlights their specific associations with ERMS. The observed complement activation, platelet involvement, and neutrophil extracellular trap (NET) formation are consistent with a pro-inflammatory, pro-thrombotic microenvironment previously reported in cancer to promote tumor growth and metastasis [68]. Interestingly, the absence of a distinct metabolite module for ERMS in WMCNA may reflect metabolic heterogeneity or limitations in metabolite detection in this subtype. In contrast, ARMS exhibited a distinct metabolite module enriched for lipid biosynthesis, GPCR signaling, and stress response pathways, consistent with its elevated anabolic demands and signaling complexity, as described above. This metabolic signature complements the proteomic findings, where ARMS-specific modules were enriched for oncogenic signaling.

On the other hand, the blue module in ARMS was enriched with immune regulatory and oncogenic signaling pathways, including mTOR, PI3K-Akt, MAPK, and HIF-1. This observation aligns consistently with more aggressive phenotypes with higher metastatic potential of this subtype over the counterpart subgroup [69]. The PI3K-Akt-mTOR axis was often hyperactivated in RMS (in this study), including key effectors such as AKT1 and AKT2. These serine–threonine kinases mediate mTORC1 phosphorylation and activate numerous downstream targets that regulate cell proliferation, survival, motility, angiogenesis, and apoptosis [70]. The marked contrast between ERMS and ARMS, where ERMS exhibited a strong immune/coagulation-related profile while ARMS showed dominance of oncogenic signaling. The divergent profiles not only reflect the distinct biological behaviors of each subtype but also emphasize the importance of developing subtype-specific therapeutic strategies.

Multi-omics integration using DIABLO and MOFA reinforced and extended single-omics findings and confirms the involvement of key biological processes, including oxidative stress response, immune modulation, ECM remodeling, cell adhesion, cell cycle regulation, and lipid metabolism. These processes are well-established hallmarks of cancer progression and therapeutic response [71,72]. The integration revealed tightly coordinated protein–metabolite networks, highlighting functional crosstalk between signaling and metabolic pathways in a subtype-specific manner.

In ARMS, a strong protein–metabolite co-regulation was noticed. Notably, proteins of cell cycle regulators such as CCNE1 and MTPN were significantly correlated with metabolites, including tetrahydrobiopterin and indole-3-carboxaldehyde. CCNE1, a critical regulator of the G1/S cell cycle transition, has been previously linked to increased disease severity, poor prognosis, and reduced chemotherapy response in osteosarcoma [73]. In our study, CCNE1 was uniquely expressed in ARMS compared to ERMS, an observation previously reported in an earlier study [74].

Tetrahydrobiopterin, an essential cofactor in nitric oxide synthesis, has been implicated in promoting tumor angiogenesis via the activation of PI3K/Akt pathway, suggesting a possible contribution to vascularization processes in ARMS [75]. Indole-3-carboxaldehyde, a metabolite derivative from tryptophan catabolism, modulates tumor progression and immune responses through activation of the aryl hydrocarbon receptor (AHR) [76]. Although its direct role in sarcomas has not yet been established, its specific association within the ARMS metabolic network observed in our study suggests involvement in tumor proliferation and immune evasion mechanisms.

In ERMS, an integrated axis involving redox regulation, immune activity, and differentiation was observed, driven by strong protein–metabolite co-regulation. This included key associations between TRIM55 and S100A11 with metabolites such as 1-methylnicotinamide and 11α-hydroxyprogesterone. TRIM55, a muscle-specific E3 ubiquitin ligase, plays a critical role in sarcomere organization and myofiber maintenance, and its expression has been linked to tumor differentiation and prognosis across cancers, supporting the retained myogenic features of ERMS [77]. S100A11, a calcium-binding protein, has been associated with inflammatory signaling and modulation of tumor proliferation and migration [78]. The presence of 1-methylnicotinamide, a key product of the NAD^+^ salvage pathway [79], together with 11α-hydroxyprogesterone, a steroid metabolite linked to immune signaling and hormone-driven cellular responses [80], suggests active metabolic coordination supporting redox balance and immunomodulation in ERMS [81].

Finally, pathway enrichment of the integrated multi-omics dataset revealed strong activation of muscle contraction, extracellular matrix remodeling, and bone maturation pathways compared to single-omics analyses. This likely reflects ARMS’s aggressiveness and associated invasive nature. On the contrary, the muscle contraction pathway aligns with ERMS’s preserved myogenic features. These findings show that multi-omics integration uncovers key subtype-specific pathway activations critical for understanding RMS biology and guiding targeted therapies.

In addition to subtype-specific profiling, our study shows that integrated proteomic and metabolomic analyses may be associated with RMS disease stage, lymph node involvement, and age. Elevated Clusterin and Keratin-6A in high-risk patients align with their roles in stress response and cytoskeletal remodeling in aggressive tumors [82,83]. Elevated glutaconic acid and methionine in intermediate-risk patients suggest disruptions in amino acid and redox metabolism commonly associated with proliferative signaling in cancer [84,85], while increased glutamic acid in high-risk patients may indicate enhanced nitrogen metabolism linked to tumor aggressiveness in this disease stage [86]. Inflammatory markers such as S100A8 and CRISP-3 showed differential expression based on lymph node status, reflecting tumor-associated inflammation and potential differences in lymphatic dissemination [87,88]. Age-dependent metabolic differences likely reflect developmental influences on RMS biology. This finding is consistent with previous sarcoma studies and needs further validation [89]. Collectively, these findings highlight the potential of integrating clinical parameters with multi-omics profiling to identify clinically relevant molecular signatures, enable precision risk stratification, and guide targeted therapies in pediatric sarcomas.

## 5. Study Limitations and Future Directions

While our study benefits from rigorous multi-omics profiling and integrative analysis, several limitations must be acknowledged. The sample size, though sufficient for detecting robust patterns, limits broader generalization, and future studies involving larger, multicentric cohorts are warranted. Additionally, while plasma proteomics and metabolomics offer valuable systemic insights, they may not fully recapitulate intratumoral biology, underscoring the need for complementary tissue-based analyses. Functional validation of the key proteins, metabolites, and pathways identified here will be essential to elucidate their roles in RMS pathogenesis and therapeutic response.

## 6. Conclusions

In conclusion, our integrative multi-omics analysis uncovered distinct molecular signatures that differentiate ERMS and ARMS, while also delineating clinically relevant molecular patterns probably associated with disease stage, lymph node involvement, and patient age. These findings provide insights into the underlying biological mechanisms driving RMS progression and highlight potential biomarkers and therapeutic targets for risk stratification and personalized treatment planning. Our study underscores the importance of multi-omics approaches in elucidating tumor heterogeneity and paves the way for more effective and individualized therapeutic strategies for RMS patients.

## Data Availability

The MS proteomics data have been deposited in the ProteomeXchange Consortium via the PRIDE repository under the dataset identifier PXD052488. The raw metabolomics data are available in the MetaboLights repository under the study ID REQ20250410209851. All codes used for data analysis and graphical visualization are available in the GitLab repository under project ID 70302961.

## Supplementary Materials

The following supporting information can be downloaded at: https://www.mdpi.com/article/10.3390/cells14141115/s1. Figure S1: Validation of the plasma albumin depletion; Figure S2: Enrichment Analysis of Differentially Expressed Proteins (DEPs) and Metabolites in Rhabdomyosarcoma Subtypes; Figure S3: The weighted gene/metabolite co-expression network analysis (WGCNA/WMCNA); Figure S4: Multi-omics integration analysis using MOFA and DIABLO; Figure Supplementary 5: OPLS-DA analyses of proteomic and metabolomics profiles stratified by clinical parameters in RMS; Table S1: Clinical data of the three groups (Alveolar (ARMS), Embryonal (ERMS) and Control (CTL)); Table S2: Venn diagram detailed for metabolomics and proteomics data; Table S3: Differentially expressed proteins (DEPS) and unique in ERMS, control and ARMS vs CTL; Table S4: Differentially expressed metabolites (DEMS) and unique in ERMS, control and ARMS vs CTL; Table S5: GO analysis of DEPs including proteins uniquely present in each subtype; Table S6: KEGG pathway analysis of DEPs including proteins uniquely present in each subtype; Table S7:Pathway enrichment of DEMs and unique metabolites using RaMP-DB and top 25 pathways for ERMS vs CTL, ARMS vs CTL and ERMS vs ARMS; Table S8: Weighted Gene Co-expression Network Analysis (WGCNA) modules Genes; Table S9: Protein class of Control group (turquoise module)-WGCNA; Table S10: KEGG pathway analysis of ARMS group (Blue module) and ERMS group (Brown module); Table S11: Key drivers of ERMS and ARMS; Table S12: Weighted Metabolite Co-expression Network Analysis (WMCNA) modules metabolites; Table S13: Enrichment analysis of ARMS group (Brown module) WMCNA; Table S14: Proteins and metabolites of component 1 and 2 from Data Integration Analysis for Biomarker Discovery using Latent Components (DIABLO); Table S15: Enrichment analysis of proteins and metabolites of component 1; Table S16: Proteins and metabolites of factor 2 from Multi-Omics Factor Analysis (MOFA) and DIABLO; Table S17: Top 20 VIP-ranked discriminatory proteins and metabolites identified by OPLS-DA across clinical subgroups in RMS.

## Abbreviations

The following abbreviations are used in this manuscript:

RMS	Rhabdomyosarcoma
ERMS	Embryonal Rhabdomyosarcoma
ARMS	Alveolar Rhabdomyosarcoma
FDR	False discovery rate
DEPs	Differentially expressed proteins
DEMs	Differentially expressed metabolites
KEGG	Kyoto Encyclopedia of Genes and Genomes
GO	Gene ontology
OPLS-DA	Orthogonal Partial Least Squares Discriminant Analysis
WGCNA	Weighted gene co-expression network analysis
WMCNA	Weighted metabolite co-expression network analysis
MOFA	Multi-Omics Factor Analysis
DIABLO	Data Integration Analysis for Biomarker Discovery using Latent Components
