# Supplementary material for: Integrative Multi-Omics Profiling of Rhabdomyosarcoma Subtypes Reveals Distinct Molecular Pathways and Biomarker Signatures
Source: Cells. 2025 Jul 20;14(14):1115. doi: 10.3390/cells14141115 (PMC12293488; doi:10.3390/cells14141115)
Supplement: Supplementary file 1 [file cells-14-01115-s001.zip › Supplementary file.pdf]

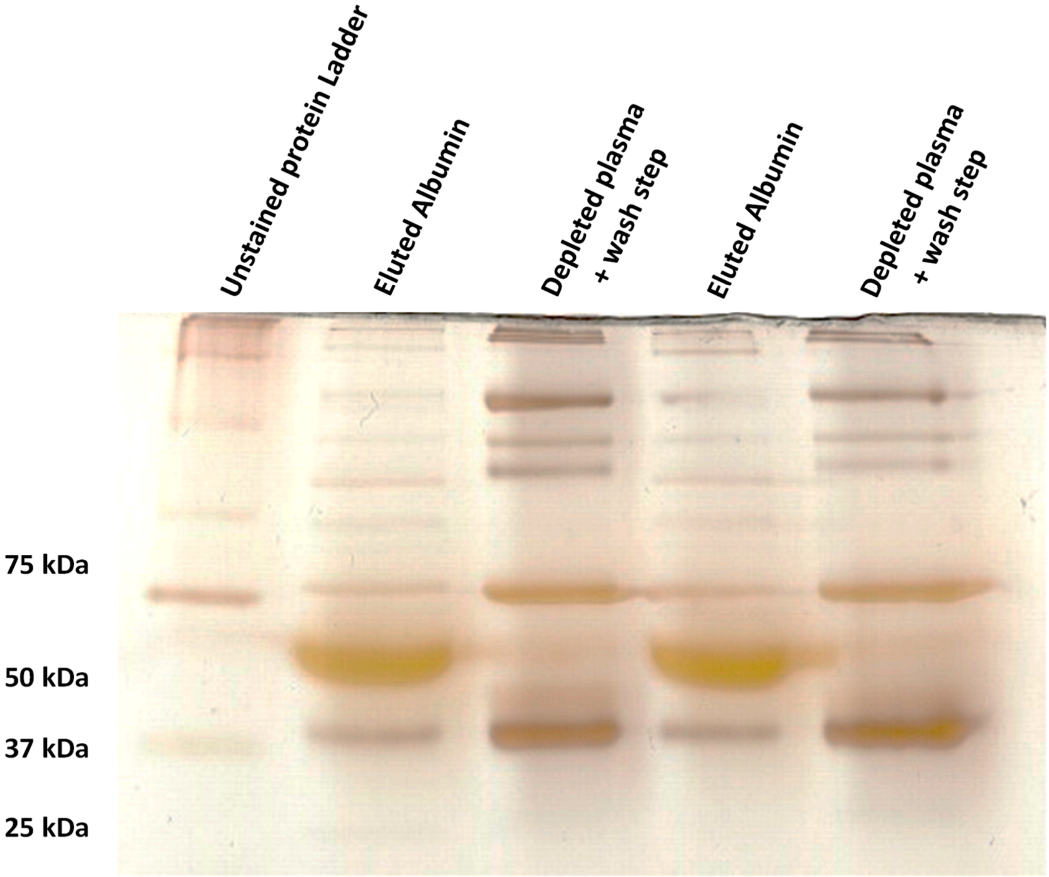

Figure Supplementary S1: Validation of the plasma albumin depletion. Silver stained SDS-PAGE gel showing two samples with an equal amount (5 µg) of depleted fraction with wash fractions, and the albumin eluted fraction using the albumin depletion kit to confirm the efficiency of depletion.

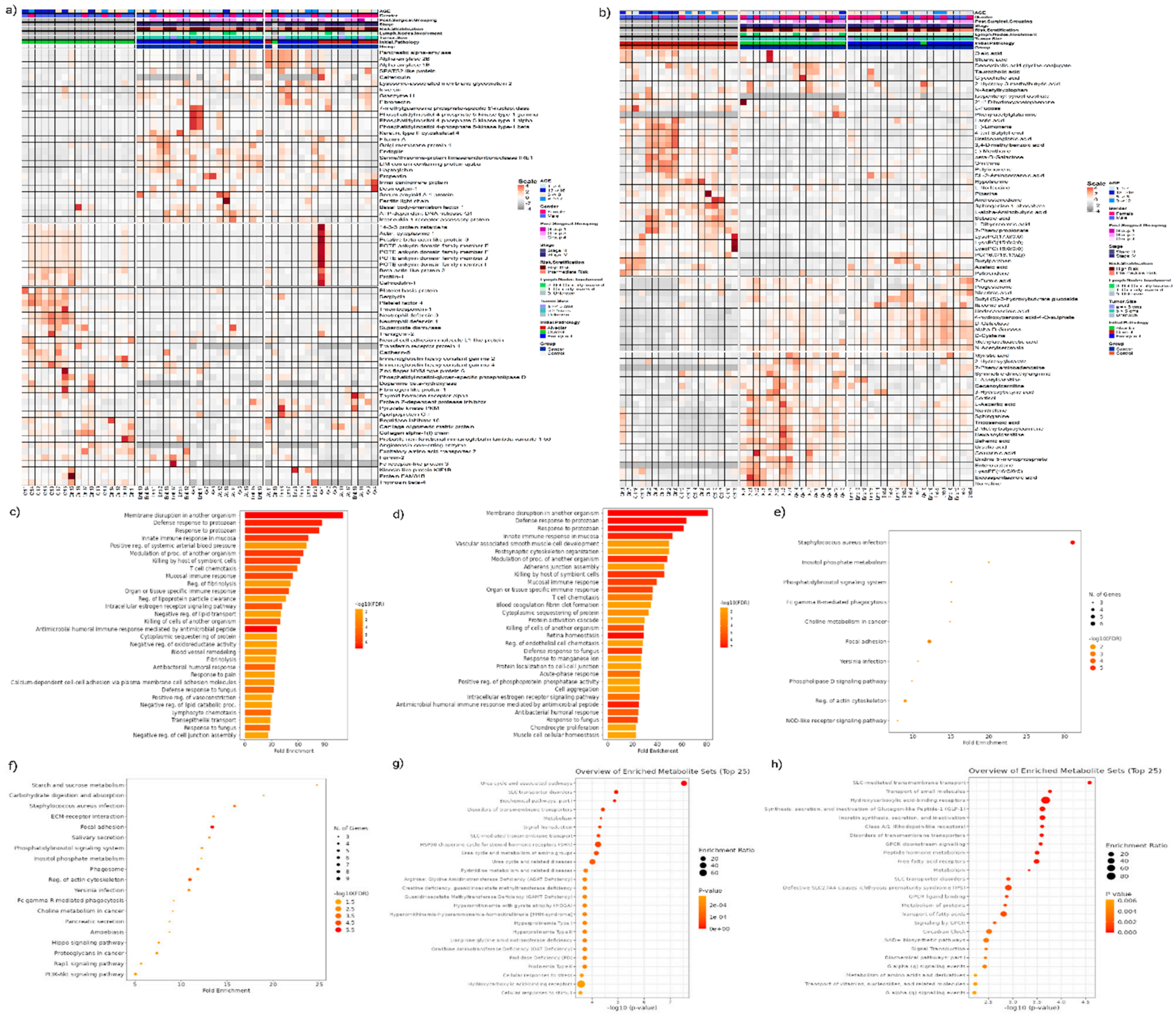

Figure Supplementary S2: **Enrichment Analysis of Differentially Expressed Proteins (DEPs) and Metabolites in Rhabdomyosarcoma Subtypes**

a) Heatmap depicting the abundance of significant proteins across the three groups, integrated with clinical data, including Age, Gender, Postsurgical group, Stage, Risk stratification, Lymph node involvement, Tumor size, and Initial pathology. **b)** Heatmap illustrating the abundance of significant metabolites in the three groups, along with associated clinical data as described above**. c)** and **d)** Bar plots illustrating the top 30 enriched biological processes of significant DEPs in ARMS/control **(c)** and ERMS/control **(d)**, as well as unique proteins for each group, sorted by fold enrichment. **e)** and **f)** Dot plots representing KEGG pathway enrichment analysis of significant DEPs in ARMS/control **(e)** and ERMS/control **(f)**, and unique proteins within each group. **g)** and **h)** Dot plots showing the top 25 enriched pathways of significant DEMs in ARMS/control **(g)** and ERMS/control **(h)**, and unique metabolites in each group, analyzed using the RaMP DB.

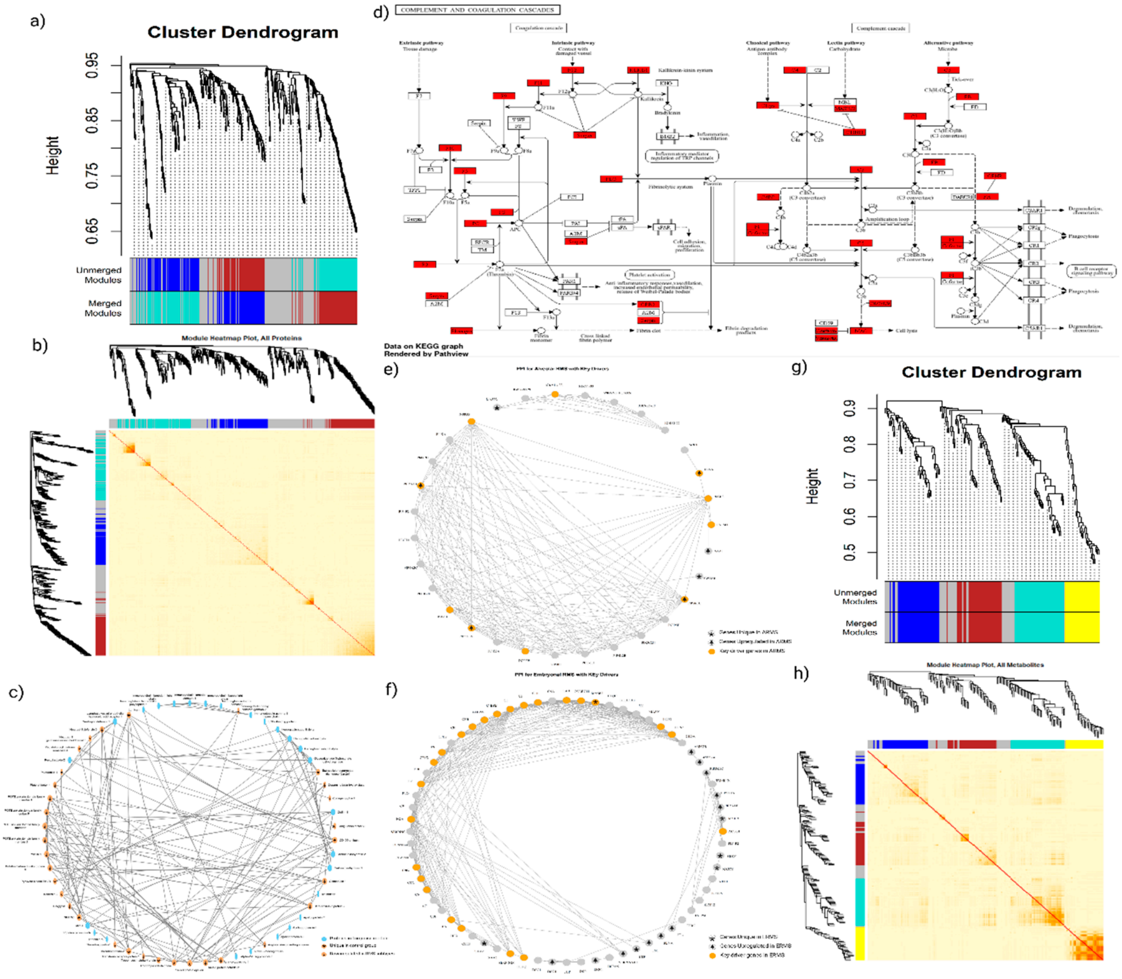

Figure Supplementary S3: The weighted gene/metabolite co-expression network analysis (WGCNA/WMCNA).

a) Protein dendrogram from hierarchical clustering with color-coded modules determined by Dynamic Tree Cut. **b)** Proteomics network heatmap with module memberships shown in color bars; saturated yellow/red indicates high co-expression. **c)** Protein network analysis for the turquoise module, strongly correlated with control samples. **d)** KEGG pathway visualization showing the role of hub, upregulated, and unique proteins from ERMS subtype (in red). **e)** Protein-protein interaction network of hub proteins in the blue module, integrated with upregulated and unique proteins identified from ARMS/Control comparisons (proteins marked with (*) shows unique proteins; arrows indicate upregulated proteins in the ARMS group). **f)** Protein-protein interaction network of hub proteins in the brown module, combined with upregulated and unique proteins identified from ERMS/Control comparisons (proteins marked with (*) shows unique proteins; arrows indicate upregulated proteins in the ERMS group). **g)** Metabolite dendrogram from hierarchical clustering with module assignment in color bars. **h)** Metabolomics network heatmap with module memberships shown; high co-expression is indicated by more saturated yellow/red colors.

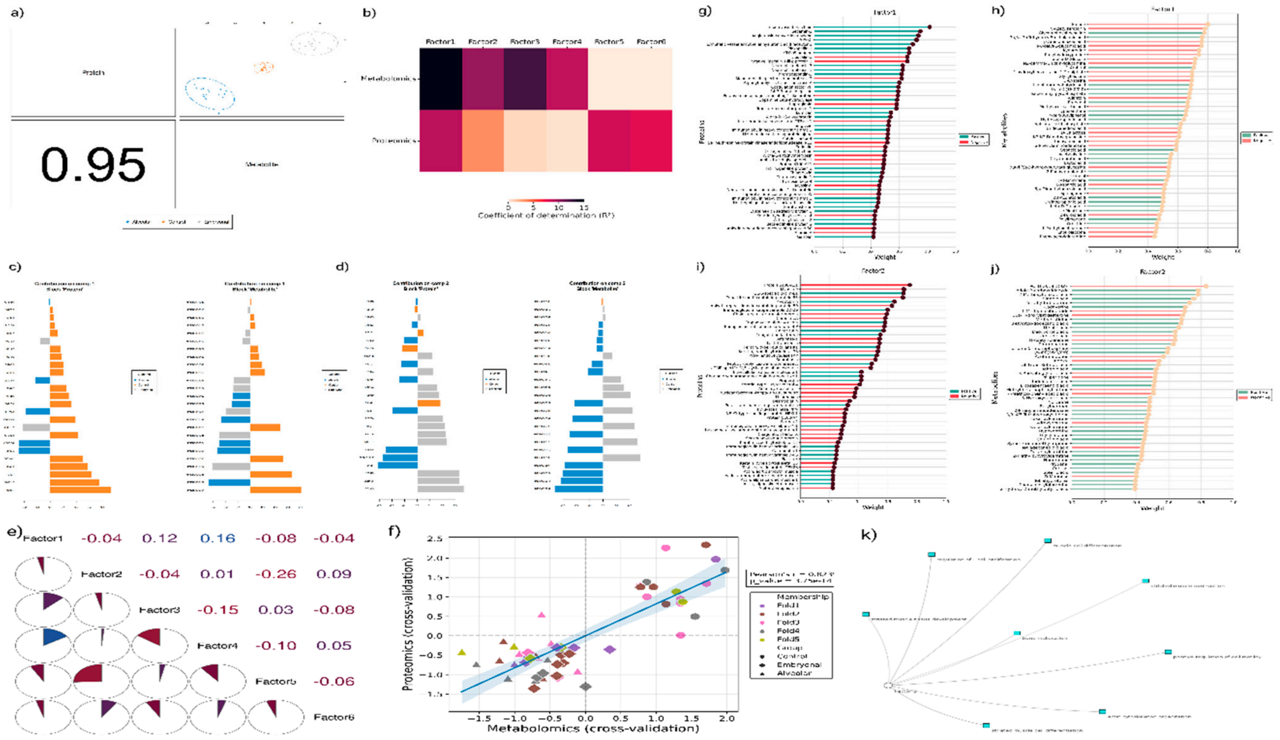

Figure Supplementary S4: Multi-omics integration analysis using MOFA and DIABLO

a) Pearson correlation analysis between proteomics and metabolomics datasets across the control, ARMS, and ERMS groups, illustrating the degree of correlation between the datasets. **b)** Bar plot illustrating the proportion of total variance (R^2) explained by factors in each of the proteomics and metabolomics datasets. **c)** Loading plot for Component 1 in DIABLO, displaying key protein and metabolite features from the supervised model, ranked by their absolute importance. Colours denote group associations: control (orange), ARMS (blue), and ERMS (grey). **d)** Loading plot for Component 2 in DIABLO, highlighting important protein and metabolite features from the supervised model, similarly ranked by absolute importance. Colours indicate group associations (control: orange, ARMS: blue, ERMS: grey). **e)** Correlation matrix of MOFA latent factors, showing the correlation coefficient between factor pairs. Positive correlations are represented in blue, while negative correlations are shown in red. **f)** Cross-validation plot of MOFA factors. The coloured bars represent the fraction of variance explained by each factor across multiple cross-validation folds. Higher bars indicate better model fit and stability of the respective factor across datasets. **g)** Weight plot showing protein contributions in MOFA's first loading, highlighting discriminative features relevant to the latent factors. **h)** Weight plot showing metabolite contributions in MOFA's first loading, emphasizing their significance within the latent factors. **i)** Weight plot illustrating protein contributions in MOFA's second loading, focusing on features significant to the latent factors. **j)** Weight plot illustrating metabolite contributions in MOFA's second loading, emphasizing their role in differentiating latent factors. **k)** Fruchterman-Reingold layout representing biological processes involving proteins from DIABLO Component 2 and MOFA Factor 2, showcasing shared functional pathways.

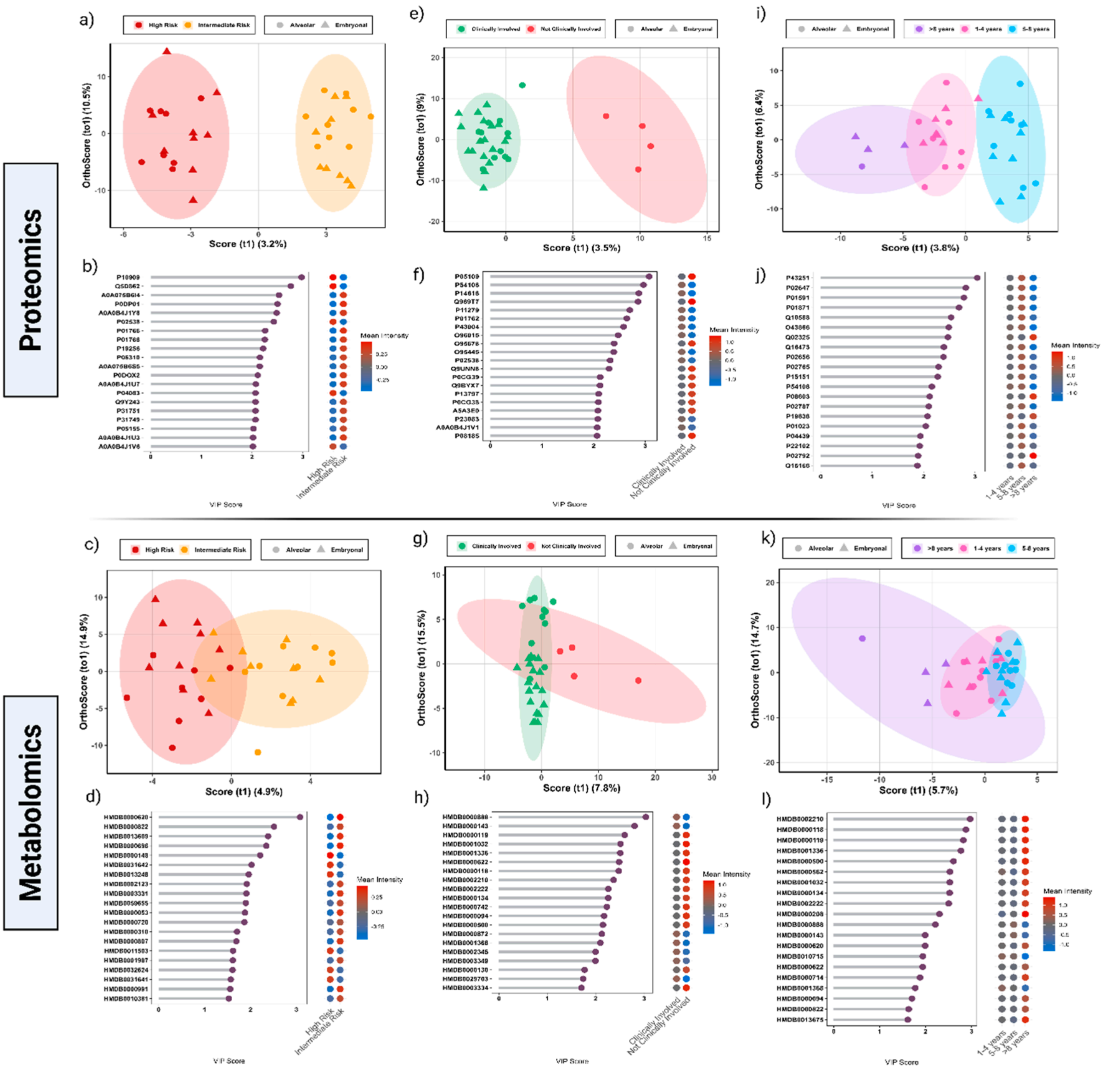

Figure Supplementary S5: OPLS-DA analyses of proteomic and metabolomics profiles stratified by clinical parameters in RMS.

a) OPLS-DA score plots showing clear separation of RMS patients by disease stage (high vs. intermediate risk) in proteomics, **b)** with the top 20 VIP-ranked discriminatory proteins. **c)** OPLS-DA plots for metabolomics with **d)** the corresponding top 20 VIP-ranked discriminatory metabolites. **e)** OPLS-DA plots demonstrating separation by lymph node involvement (yes vs. no) in proteomics, **f)** with the top 20 VIP proteins, and **g)** in metabolomics with **h)** the top 20 VIP metabolites. **i)** Age stratification (1–4, 5–8, >8 years) in proteomics OPLS-DA plots with **j)** the top 20 VIP proteins, and **k)** corresponding metabolomics OPLS-DA plots with **l)** the top 20 VIP metabolites, highlighting clear molecular differences across age groups.
